# SARS-CoV-2 ORF8: an accessory protein at the interface of immune evasion and inflammation

**DOI:** 10.3389/fimmu.2026.1826664

**Published:** 2026-08-26

**Authors:** Carolina Schäfer, Cristopher Blamey, Rocío Balbiano, Javier Mena, Claudio Acuña-Castillo, Ana María Sandino

**Affiliations:** Centro de Biotecnología Acuícola, Facultad de Química y Biología, Universidad de Santiago de Chile, Santiago, Chile

**Keywords:** endoplasmic reticulum stress, IFN pathway, immune evasion, major histocompatibility complex class I, ORF8, SARS-CoV-2, virokine

## Abstract

SARS-CoV-2 ORF8 is a rapidly evolving accessory protein that modulates host immunity through both intracellular and extracellular mechanisms. Intracellularly, ORF8 disrupts antigen presentation by reducing cell-surface MHC-I and is linked to endoplasmic reticulum remodeling and altered stress responses. Extracellularly, secreted ORF8 behaves as a virokine that can amplify inflammatory programs in myeloid and dendritic cells, with potential implications for acute severity and tissue-specific pathology. ORF8 is also reported to antagonize type I interferon induction through effects on IRF3-dependent pathways. Clinically, circulating ORF8 has been associated with disease severity, and persistent detection of ORF8 after viral RNA becomes undetectable has been reported in some individuals with post-acute symptoms. However, whether this persistence reflects a direct pathogenic role or instead marks ongoing viral burden and immune activation remains unresolved. In this review, we summarize recent findings on the pleiotropic effects of ORF8 and outline key priorities to clarify when ORF8 acts primarily as an immune-evasion factor, an inflammatory amplifier, or both. Collectively, the evidence reviewed here identifies ORF8 as a promising candidate for further mechanistic studies, whose biomarker potential and therapeutic relevance warrant rigorous evaluation in acute and post-acute COVID-19.

## 1 Introduction

Coronavirus disease 2019 (COVID-19) has resulted in more than 7 million officially reported deaths worldwide (1). Although the World Health Organization declared an end to COVID-19 as a public health emergency in May 2023 (2), its etiologic agent, severe acute respiratory syndrome coronavirus 2 (SARS-CoV-2), continues to circulate globally, representing a public health threat due both to the acute illness it causes and to the persistent syndrome known as Long COVID (3). Beyond typical respiratory manifestations, SARS-CoV-2 can induce immune dysfunction, including chronic inflammation, immune evasion via alterations in antigen presentation and T-cell exhaustion, factors linked to increased disease severity (4).

Accessory proteins have emerged as key effectors underpinning the immune dysregulation described above, as they may influence disease pathogenesis through a wide range of mechanisms. Several SARS-CoV-2 accessory proteins alter innate immune and inflammatory pathways: ORF3a promotes inflammasome activation and apoptosis (5, 6), ORF6 blocks interferon signaling by disrupting nucleocytoplasmic transport (7) and ORF9b suppresses mitochondrial antiviral signaling (8). A subset of these proteins, ORF3a, ORF7a and ORF8, has also been reported to participate in the downregulation of MHC-I, thereby impairing antigen presentation to cytotoxic CD8^+^ T cells, albeit through different mechanisms (9, 10). Among these immune-modulatory proteins, ORF8 is distinguished by its high genetic variability and its interactions with a broad array of host molecules, primarily those involved in vesicular trafficking and protein quality control in the endoplasmic reticulum (ER) (11, 12). ORF8 has also been implicated in exacerbating SARS-CoV-2–induced inflammation and, because it is a secreted protein, it has been proposed to act as a virokine (11, 13). This apparent proinflammatory role contrasts with reports of type I interferon (IFN) antagonism, suggesting that ORF8 may function as a complex immune modulator with context-dependent functions (14). Together, these features distinguish ORF8 from other SARS-CoV-2 accessory proteins and make it a compelling focus for further mechanistic studies.

Interestingly, ORF8 is among the least conserved proteins across the *Coronaviridae* family: a clear ortholog of the SARS-CoV-2 ORF8 protein has not been identified in non-sarbecovirus human coronaviruses, including MERS-CoV and endemic HCoVs (15). Even among sarbecoviruses, SARS-CoV-2 ORF8 exhibits only limited sequence homology to SARS-CoV-1 ORF8ab (16). Both SARS-CoV-2 ORF8 and SARS-CoV-1 ORF8ab have been shown to induce ER stress and activate the unfolded protein response, which can prevent apoptosis and favor viral replication (15, 17). However, only SARS-CoV-2 ORF8 has demonstrated the ability to reduce cell-surface MHC-I expression, a novel immune-evasion strategy likely acquired through a mutagenic event and therefore the focus of multiple studies (10, 18, 19). Moreover, the structural features of ORF8 and its ability to be secreted from infected cells raise the possibility that its effects may extend far beyond the site of initial infection.

**Figure 1:**
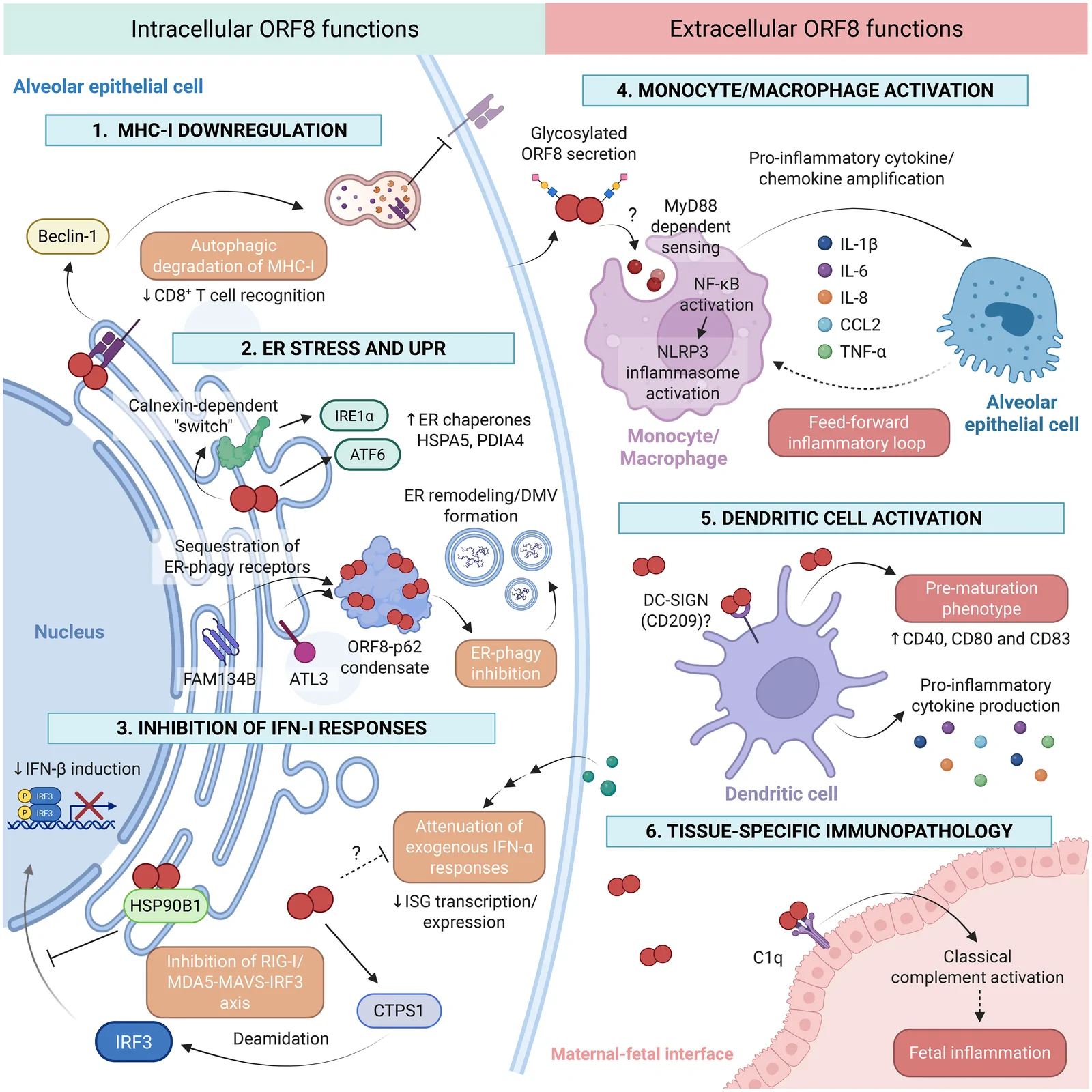
Mechanistic overview of the intracellular and extracellular immunomodulatory functions of SARS-CoV-2 ORF8. ORF8 has been reported to modulate host immunity through multiple intracellular and extracellular mechanisms. 1) MHC-I downregulation: intracellular ORF8 promotes Beclin-1-dependent autophagic degradation of MHC-I, reducing its cell-surface abundance and consequently limiting recognition by cytotoxic CD8^+^ T cells. 2) ER stress and unfolded protein response (UPR): ER-localized ORF8 interacts with calnexin and induces a calnexin-dependent stress response involving predominantly the IRE1α/XBP1 branch, while ORF8-induced ER stress has also been associated with ATF6 activation and increased expression of ER chaperones, including HSPA5 and PDIA4. In parallel, ORF8 forms condensates with p62/SQSTM1 that sequester the ER-phagy receptors FAM134B and ATL3, inhibiting ER-phagy and favoring ER remodeling and double-membrane vesicle (DMV) formation. 3) Inhibition of type I IFN responses: ORF8 suppresses IFN-I induction through effects on the RIG-I/MDA5-MAVS-IRF3 axis, including mechanisms involving HSP90B1 and CTPS1-mediated IRF3 deamidation. ORF8 can additionally attenuate cellular responses to exogenous IFN-α and reduce interferon-stimulated gene (ISG) expression, although the molecular basis of this downstream effect remains unresolved. Extracellularly, secreted ORF8 can act as an immunomodulatory virokine. 4) Monocyte/macrophage activation: ORF8 induces NF-κB-associated inflammatory signaling and promotes NLRP3 inflammasome activation, resulting in increased production of cytokines and chemokines such as IL-1β, IL-6, IL-8, CCL2, and TNF-α. MyD88-dependent sensing has also been implicated in ORF8-induced myeloid activation, although the upstream receptor remains undefined. These responses may contribute to a proposed feed-forward inflammatory circuit between macrophages and alveolar epithelial cells. 5) Dendritic-cell activation: ORF8 directly induces a pre-maturation phenotype and proinflammatory cytokine production in dendritic cells. DC-SIGN/CD209 has been identified as an ORF8-binding partner and proposed as a candidate receptor, although its requirement for the dendritic-cell phenotype has not been established. 6) Tissue-specific immunopathology: at the maternal-fetal interface, ORF8 binds C1q and promotes classical complement activation in trophoblast models; complement activation and inflammatory responses in fetal compartments have been associated with ORF8 exposure, although the causal relationship between these processes remains to be fully established. Solid arrows denote experimentally supported functional or physical relationships, whereas dashed lines and question marks indicate proposed, indirect, or mechanistically unresolved connections. Created in BioRender (65).

Of note, much of the mechanistic evidence discussed in this review derives from overexpression systems, recombinant protein assays, or in silico approaches, which may not fully recapitulate ORF8 biology at the protein levels and in the molecular contexts present in naturally infected cells. Therefore, we distinguish experimentally validated mechanisms from computational predictions and note where the physiological relevance of a given model is unclear. With this framework in mind, we summarize the latest findings regarding ORF8 and its effects on the host immune response and inflammatory pathways, and provide an integrated overview of its functions, proposed mechanisms, and the current level of supporting evidence (**Table 1**, **Figure 1**).

**Table 1:** Reported functions of SARS-CoV-2 ORF8, proposed mechanisms, and current level of supporting evidence.

Reported ORF8-associated functions	Proposed mechanism	Evidence type	Main limitation/comment	Key references
Modulation of Spike abundance	Host protein synthesis inhibition and ER retention of Spike, likely via ORF8-Spike interaction, reducing virion incorporation and recognition by anti-Spike antibodies.	*In vitro*	Supported in cell-based systems, but physiological relevance and overall consequence for transmissibility remain unresolved	Chou et al. (26); Kim et al. (27)
MHC-I downregulation	Beclin-1–dependent autophagic degradation of MHC-I	*In vitro*	Functional effect supported, but not all intermediate steps have been independently confirmed across systems	Zhang et al. (10)
	Differential reduction of HLA-A, HLA-B and HLA-C surface expression, with more pronounced downregulation of HLA-C; associated with enhanced NK-cell degranulation	*In vitro*; clinical genetic association	Locus-dependent effects were demonstrated in model systems, but variability across individual HLA-I allotypes and naturally infected primary cells remains unresolved	Wang et al. (37)
	Requirement for ER localization and ORF8 signal peptide	*In vitro*	Supports ER-linked mechanism but does not by itself prove direct ORF8–MHC-I binding	Matsuoka et al. (18); Arshad et al. (9)
	Direct binding of ORF8 to MHC-I through specific interfaces	*In silico*	Computational prediction only; direct interaction remains experimentally unvalidated	Cheng and Peng (32); Wu et al. (33); Chaudhari et al. (34)
	Interference with calnexin/PDI/PLC network, biasing MHC-I toward ER retention or degradation	*In vitro*; indirect mechanism inferred	Plausible model, but direct causal link to MHC-I surface loss remains incomplete	Gordon et al. (12); Wang et al. (36); Liu et al. (41)
ER stress and UPR modulation	Activation of ATF6 and IRE1 branches	*In vitro*	Branch selectivity may vary by system	Rashid et al. (42)
	Predominant IRE1α/XBP1 activation with calnexin-dependent effects	*In vitro*	May reflect cell-type or species-specific context	Wang et al. (36)
	ER-phagy blockade through p62 condensates that sequester FAM134B and ATL3	*In vitro*	Physiological relevance during natural infection requires further validation	Tan et al. (43)
	ER remodeling through mixed disulfides with ER oxidoreductases	*In vitro*	Mechanistically compelling, but link to all downstream phenotypes remains unresolved	Liu et al. (41)
Proinflammatory activity/virokine-like role	Monocyte/macrophage cytokine induction via NF-κB-linked signaling	*In vitro* and *ex vivo*	Initiating receptor remains uncertain	Lin et al. (45)
	NLRP3 inflammasome activation after ORF8 uptake in monocytes	Patient-derived samples plus *in vitro* assays	Uptake route and upstream receptor remain unresolved	Wu et al. (31)
	DC-SIGN-mediated activation of dendritic cells	*In vitro*	Needs broader confirmation in primary human systems	Hamdorf et al. (46)
	IL-17RA/RC engagement	*In vitro*; debated	Conflicting evidence; functional relevance in myeloid cells is uncertain	Wu et al. (21); Lin et al. (45)
	MyD88-dependent sensing independent of IL-17 receptor	*In vitro*	Supports alternative proximal sensing pathway, but exact receptor remains unidentified	Ponde et al. (47)
	Complement activation at the maternal-fetal interface through binding to C1q	Tissue model/translational	Supports tissue-specific immunopathology, but evidence is currently limited to a specialized biological context	Azamor et al. (48)
	Inflammatory and osteoclastogenic effects by increasing mediators such as TNF, IL6, CCL2, and the RANKL/OPG ratio	Clinical association plus *ex vivo* assays	Supports a pathogenic role for ORF8 in a defined inflammatory context, but generalizability beyond IMID-associated bone pathology remains uncertain	Melano et al. (49)
	Glycoform-dependent inflammatory activity and unconventional secretion via YIF1B	*In vitro*	Effects appear system-dependent and need standardization	Lin et al. (50); Lin et al. (51)
Type I IFN antagonism	Reduced IRF3 nuclear translocation and IFN-β induction	*In vitro*	Based largely on overexpression/poly(I:C) systems	Rashid et al. (42)
	HSP90B1/GRP94-linked inhibition of RIG-I/MDA5-MAVS-IRF3 signaling	*In vitro*	Relative contribution versus other reported mechanisms is unclear	Chen et al. (52)
	CTPS1-mediated deamidation of IRF3	*In vitro*, mechanistic evidence	Promising mechanism, but independent confirmation is still limited	Rao et al. (53)
	Suppression of IFN-α-induced ISRE activity and ISG expression without preventing STAT1/STAT2 nuclear translocation	*In vitro*	Indicates inhibition downstream of IFN-I induction, but the molecular target responsible for impaired ISG transcription remains undefined	Lu et al. (55)
	Residues 51-56 (ARKSAP) contribute to suppression of IFN-α-induced ISRE activity	*In vitro*; recombinant SARS-CoV-2	Molecular mechanism linking the ARKSAP region to downstream IFN-I responses remains unresolved	Zhou et al. (56)

## 2 Epidemiological and clinical relevance of ORF8

The genomic region encoding ORF8 is highly prone to mutation. The L84S substitution, one of the most common ORF8 variants, was identified early in the pandemic and has been associated with reduced disease severity (20). *In vitro*, the L84S variant displays attenuated pro-inflammatory activity, including reduced cytokine induction in monocyte-based systems, providing a possible mechanistic explanation for the milder clinical outcomes observed in patients infected with this variant (21, 22). Similarly, several Omicron-derived lineages have acquired mutations that reduce or abrogate ORF8 expression. In XBB.1.5, predominant in 2023, a premature stop codon truncated ORF8; infection with this lineage was associated with attenuated pulmonary pathology in hamster models, consistent with the broader reduction in disease severity observed for several Omicron-derived viruses (23). Together, these findings support the idea that ORF8 can contribute to pathogenic phenotypes in specific experimental contexts.

However, the epidemiological consequences of ORF8 loss are complex. Although ORF8 truncation has been proposed to confer an adaptive advantage and increase transmissibility of SARS-CoV-2 (24), this interpretation remains difficult to disentangle from the effects of the broader genomic background of each lineage, particularly Spike mutations that strongly shape receptor engagement, entry efficiency, antigenicity and lineage expansion (25). Mechanistically, ORF8 expression has been reported to cause dose-dependent downregulation of Spike and incomplete S1/S2 cleavage, resulting in reduced Spike protein display on the surface of virions and diminished infectivity *in vitro* (26, 27). Therefore, reduced ORF8 expression or stability could, in some settings, relieve this constraint on Spike maturation and virion infectivity. Clinically isolated ORF8 variants with reduced protein stability showed attenuated Spike-suppressive activity and enhanced Spike-mediated infectivity *in vitro*, while preserving their inhibitory effects on MHC-I (26). However, because these findings derive from overexpression and pseudovirus systems, and because clinical lineages carry multiple co-occurring mutations, they should be interpreted as mechanistic support for a hypothesis rather than direct evidence that ORF8 loss increases transmission *in vivo*.

The evolutionary history of ORF8 further supports a context-dependent model. Although ORF8-truncated lineages have emerged repeatedly, they have not persisted in global circulation. Subsequent large infection waves have been driven by variants with intact ORF8, such as BA.2.86 and its descendant JN.1. Moreover, several recently circulating lineages (e.g. XEC and LP.8.1) also descend from BA.2.86 and retain an intact ORF8 (28–30). This pattern indicates that ORF8 loss is not required for lineage expansion and that ORF8 retention can remain compatible with viral fitness. Whether ORF8 conservation confers a critical advantage through immune evasion, or reflects linkage with favorable Spike mutations remains unsolved (24). Disentangling these variables will require experimental systems in which ORF8 status is varied against a fixed Spike genomic background.

Clinically, serum ORF8 levels have been found to correlate with disease severity and mortality in newly hospitalized patients (31). In a subset of individuals with Long COVID, ORF8 was detected across serial samples for more than a year, indicating persistent antigenemia alongside persistent symptoms (31). Whether this association reflects a direct pathogenic role for ORF8 or instead its value as a biomarker of ongoing viral burden in post-acute disease remains to be established.

## 3 Antigen presentation disruption by ORF8

Downregulation of surface MHC-I is a widespread immune-evasion strategy employed by diverse viruses to limit antigen presentation and escape recognition by cytotoxic CD8^+^ T cells. SARS-CoV-2 appears to have converged on this same strategy through multiple accessory proteins. The viroporin-like transmembrane protein ORF3a reduces MHC-I surface expression by disrupting trafficking through the secretory pathway, whereas ORF7a has been reported to bind the MHC-I heavy chain and impede its export from the ER (9). ORF8, the focus of this section, appears to act primarily through post-translational, degradative routes, targeting MHC-I for autophagic-lysosomal degradation (10). This phenomenon has been demonstrated by experimental studies in cell-based systems, although the precise molecular route responsible for MHC-I loss remains incompletely defined. Available data suggest that this effect is linked to the ER localization of ORF8 (9, 10), as its signal peptide is required for efficient MHC-I downregulation (18). Zhang et al. (10) proposed that ORF8 promotes Beclin-1-dependent autophagic degradation of MHC-I, thereby depleting intracellular pools and reducing cell-surface expression. However, definitive experimental evidence is still lacking to determine whether direct binding of ORF8 to MHC-I is responsible for its downregulation at the cell surface, and to identify the ORF8 domains that mediate this putative interaction (18).

To date, multiple *in silico* studies have proposed candidate ORF8/MHC-I interaction surfaces by mapping potential binding interfaces through molecular docking and structural analysis (14, 32). Several works have associated conserved complementarity-determining region (CDR)-like motifs in ORF8 with candidate MHC-I–binding sites. For example, Wu et al. (33) defined CDR1 (residues 51–55), CDR2 (62–75), and a CDR3-like loop (104–110), proposing that interaction with MHC-I may occur through the CDR2- and CDR3-like loops. By contrast, Cheng and Peng et al. (32) identified three conserved segments in the ORF8 structure (39–42, 104–107, and 110–112) and proposed the following residues as potential contact sites with MHC-I: 40, 104, 105, 106, 107, and 110. Additional docking analyses have suggested that mutations in the delta variant disrupting ORF8 dimerization may reduce MHC-I interaction (34), but these computational models remain unvalidated experimentally. Importantly, Moriyama et al. (35) experimentally tested the impact of six naturally occurring, nonsynonymous ORF8 mutations retaining detectable ORF8 protein expression (T11I, I121L, L84S, E92K, Δ119–120, and V100L), and found that none reversed MHC-I downregulation suggesting that these residues do not participate in the interaction with MHC-I or that the substitutions are insufficiently destabilizing. Consequently, fundamental questions remain regarding the structural determinants of ORF8 that mediate this function.

A growing body of experimental evidence places ORF8 in direct contact with the ER chaperone network that supports MHC-I folding and peptide loading (11, 12, 36). ORF8 has been reported to engage calnexin, a key mediator of heavy-chain folding and glycan quality control, and, in some systems, to contact ER oxidoreductases of the PDI family, providing plausible routes to perturb assembly of the peptide-loading complex (PLC), redox-assisted folding, and cargo hand-off (36). Such interference would be expected to bias MHC-I toward ER retention or ER-associated degradation, thereby reducing its surface density and contributing to impaired antigen presentation, independently of a putative direct ORF8-MHC-I interaction.

Several important questions regarding the immunological consequences of ORF8-mediated MHC-I downregulation remain underexplored. First, although ORF8 was initially characterized mainly through total MHC-I or HLA-A2/HLA-A*02:01 readouts, subsequent work using locus- or allotype-specific antibodies showed that SARS-CoV-2 ORF8 can reduce surface expression of HLA-A, HLA-B, and HLA-C in model cell systems (37). Notably, Wang et al. observed a more pronounced ORF8-mediated downregulation for HLA-C than for HLA-A/B, suggesting that ORF8 does not affect all HLA-I molecules uniformly. However, the extent to which this variation applies across individual HLA-I allotypes remains unresolved (9, 10). This question is particularly relevant because HLA-I allotypes differ in peptide-loading requirements, tapasin dependence, intracellular trafficking, surface stability, and peptide repertoire (38, 39). Second, the magnitude of ORF8-mediated MHC-I loss may also vary by cell type, since most mechanistic studies have relied on HEK293T-based or selected epithelial cell systems (10), whereas naturally infected airway epithelial, endothelial, myeloid, or other immune-cell populations may differ in ORF8 abundance, ER quality-control machinery, antigen-processing capacity, and baseline MHC-I turnover. Finally, downregulation of classical MHC-I is a double-edged strategy: while it impairs recognition of infected cells by cytotoxic CD8^+^ T lymphocytes, it may simultaneously expose those cells to natural killer (NK) cell-mediated lysis through “missing-self” recognition. Consistent with this, ORF8-mediated HLA class I downregulation has been associated with increased NK-cell degranulation in model systems, and individuals carrying HLA/KIR genotypes that favor NK activation upon MHC-I loss have been associated with reduced COVID-19 severity (37). This balance may be further influenced by non-classical molecule HLA-E, which has been observed to be spared or even upregulated during infection: preserved HLA-E may allow recognition by HLA-E-restricted CD8^+^ T cells despite the loss of classical HLA-Ia (40). Together, these observations indicate that the immune-evasion benefit ORF8 confers by lowering classical MHC-I may be partly offset by heightened NK-cell and non-classical T-cell recognition. Thus, the net effect of ORF8 on immune surveillance *in vivo* remains to be defined.

## 4 ER stress and the unfolded protein response

SARS-CoV-2 ORF8, like its homolog from SARS-CoV-1, modulates ER proteostasis by linking viral replication to ER stress and activation of the unfolded protein response (UPR). Studies in cell-based systems have documented ORF8 accumulation at the ER, alterations in ER architecture, and enhanced double-membrane vesicle (DMV) formation; loss or mutation of ORF8 blunts these effects, consistent with a direct role for ORF8 in remodeling ER homeostasis (41, 42).

ORF8 has been reported to induce ER stress and selectively activate the UPR via the ATF6 and IRE1 branches, without robust engagement of the PERK arm. These findings align with the ER localization of ORF8 and the upregulation of canonical UPR target genes, supporting a model in which ORF8 perturbs proteostasis to reshape host cell physiology (42). Similarly, Wang et al. (36) identified an ER-stress–like program acting predominantly via the IRE1α/XBP1 branch, with strong induction of ER chaperones including BiP/HSPA5 and PDIA4. They further proposed a calnexin-dependent regulatory switch that accounts for stronger UPR responses in human compared with murine cells and facilitates viral replication. A complementary mechanism was described by Tan et al. (43), who showed that ORF8 forms condensates with the autophagic cargo receptor p62 (SQSTM1) that sequester the ER-phagy receptors FAM134B and ATL3, thereby inhibiting ER-phagy; the resultant accumulation of ER cargo likely amplifies ER stress and promotes DMV biogenesis. Collectively, these data support a model in which ORF8 perturbs ER homeostasis through direct effects on UPR sensors and indirect effects via ER-phagy blockade, with the precise branch readouts dependent on cell type, species, and experimental context.

## 5 Proinflammatory role of ORF8

As a secreted, N-glycosylated, dimeric protein with an immunoglobulin-like fold, ORF8 has been proposed to act as a virokine that potentiates inflammatory responses (44). Multiple studies support a role for secreted ORF8 in amplifying innate inflammation. Recombinant, glycosylated ORF8 stimulates TNF-α, IL-1β, IL-6, and CCL2 production in human monocytes/macrophages via NF-κB-linked signaling, consistent with clinical associations between circulating ORF8 and severe disease (45). In CD14^+^ monocytes, internalized ORF8 has been shown to bind NLRP3 and promote inflammasome activation, leading to IL-1β, IL-8, and CCL2 release, although whether the uptake route is receptor-mediated or phagocytic remains unresolved (31). In dendritic cells, ORF8 has been reported to drive a pre-maturation phenotype with broad cytokine and chemokine induction, with the C-type lectin DC-SIGN (CD209) proposed as a candidate receptor, based on binding and co-immunoprecipitation assays (46).

Nevertheless, the identity of the initiating receptor(s) in myeloid cells remains a matter of debate. Early proteomic maps and follow-up studies proposed IL-17RA/RC engagement; however, IL-17RC is scarce in monocytes/macrophages, and ORF8-induced cytokines persist in IL-17RA-deficient or antibody-blocked systems, arguing against a functional IL-17 axis in these cells. Instead, MyD88 dependence points to TLR-family involvement as a more plausible explanation for ORF8-driven myeloid activation (12, 31, 47). Thus, while ORF8 behaves as a virokine, the proximal sensing machinery appears to be context-dependent and is not yet definitively established.

The inflammatory footprint of ORF8 extends to specialized tissues. At the maternal-fetal interface, ORF8 crosses the placenta and binds C1q, triggering classical-pathway complement activation in trophoblast models (48). Notably, fetal compartments with detectable ORF8 exhibit heightened inflammatory signatures even when viral RNA is undetectable, and maternal ORF8 can persist for more than 200 days, linking persistent antigenemia to prolonged symptoms (48). In addition, recent evidence indicates that circulating ORF8 may exacerbate inflammatory responses and osteoclastogenesis in patients with preexisting immune-mediated inflammatory diseases such as rheumatoid arthritis, further supporting tissue-specific pathological effects of secreted ORF8 (49). Secretion biology appears to modulate these effects. Conventionally secreted ORF8 is N-glycosylated and dimeric, whereas unglycosylated ORF8 can be released via an unconventional, YIF1B-mediated route; some studies suggest that glycosylation state influences receptor engagement and NF-κB activation, although results vary by system and require further standardization (50, 51).

Together, these data suggest a model in which secreted ORF8 acts as an immunomodulatory ligand that can prime or exacerbate myeloid and dendritic-cell responses through NLRP3 activation and putative pattern-recognition receptors, with tissue-specific manifestations. However, much of this evidence derives from recombinant protein and overexpression systems, and key aspects, such as the identity of the entry receptors, the functional role of distinct glycoforms, and *in vivo* dose-response relationships, remain to be clarified before its contribution to COVID-19 immunopathology can be firmly established.

## 6 Type I IFN pathway inhibition

Beyond its reported proinflammatory activities, ORF8 has paradoxically been reported to antagonize type I IFN induction (14). Several overexpression studies converge on IRF3 as a principal node targeted by ORF8. Rashid et al. (42) described that, upon poly(I:C) stimulation, both L84 and S84 ORF8 variants reduce IFN-β transcription and downstream ISG induction (e.g., ISG15, ISG56), coincident with diminished IRF3 nuclear translocation. Complementarily, Chen et al. (52) proposed that the interaction of ORF8 with the ER chaperone HSP90B1 (GRP94), a factor linked to IRF3 activity, contributes to the ORF8-mediated inhibition of the RIG-I/MDA5-MAVS-IRF3 axis. More recently, Rao et al. (53) reported that ORF8 engages CTPS1 to promote IRF3 deamidation, rendering it transcriptionally incompetent while simultaneously enhancing CTP biosynthesis, thus inhibiting IFN production and supporting nucleotide availability for viral replication. Nevertheless, the net effect of ORF8 *in vivo* appears more complex: deletion of ORF8 in murine models using recombinant SARS-CoV-2 has been shown to exacerbate lung inflammation without increasing viral load, suggesting a context-dependent immunomodulatory role for ORF8 (54).

In addition to suppressing IFN-I induction, ORF8 may also interfere with cellular responses to exogenous IFN. Lu et al. (55) used an interferon-stimulated response element (ISRE) reporter assay and found that ORF8 reduced IFN-α-induced reporter activity and the expression of several ISGs, including IFIT3 and IFITM1-3, without preventing STAT1 or STAT2 nuclear translocation. More recently, Zhou et al. (56) found that deletion of residues 51–56 (ARKSAP) from ORF8 in replication-competent SARS-CoV-2 partially relieved suppression of IFN-α-induced ISRE activity. This motif was originally proposed by Kee et al. (57) to mimic an ARKS motif in histone H3 and thereby perturb host chromatin regulation; however, the nuclear localization and histone-mimic function of ORF8 have subsequently been challenged (58). Thus, although the findings of Zhou et al. implicate the ARKSAP region in modulation of downstream IFN-I responses, whether this effect is mechanistically related to chromatin regulation remains unresolved. Taken together, these studies delineate multiple, potentially complementary routes by which ORF8 may suppress IFN-I responses, including IRF3-dependent IFN production and downstream responses to exogenous IFN. Although the latter phenotype has now been supported in a recombinant-virus system, its underlying molecular mechanism remains unresolved, and the relative contribution of these pathways during infection remains incompletely defined.

It is worth noting that the proinflammatory and IFN-antagonizing activities of ORF8 are not necessarily mutually exclusive: A plausible model is that intracellular ORF8 in infected cells suppresses IRF3-dependent antiviral IFN-I induction, whereas secreted or extracellular ORF8 can stimulate NF-κB-, MyD88-, or inflammasome-linked cytokine programs in bystander myeloid and dendritic cells. In this framework, ORF8 would not simply be pro- or anti-inflammatory, but rather would function as a context-dependent modulator that uncouples antiviral IFN responses from inflammatory cytokine production. Such functional decoupling has also been described for other SARS-CoV-2 proteins, including nsp6 and nsp14 (59, 60). However, studies that directly quantify ORF8-mediated IFN antagonism and inflammatory cytokine induction in the same experimental system under physiologically relevant conditions are currently lacking; the net outcome will likely depend on cell type, infection stage, and ORF8 concentration.

## 7 Discussion

ORF8 is a pleiotropic modulator of host immunity with a set of features that make it a particularly compelling focus for further investigation. Its rapid evolutionary rate suggests that ORF8 has been repeatedly reshaped during host switching and adaptation, raising the possibility that its current properties in SARS-CoV-2, particularly MHC-I modulation and secretion as a virokine, represent relatively recent functional specializations that may contribute to the specific immunopathological profile of COVID-19.

Intracellularly, ORF8 integrates three broad classes of activity: remodeling of ER proteostasis, antagonism of type I IFN pathways, and interference with antigen presentation. Together, these functions may favor viral fitness by dampening antiviral responses and limiting cytotoxic T cell-mediated killing of infected cells. However, whether these activities are sufficient to promote prolonged infection or tissue persistence in immunocompetent hosts remains unresolved. *In vitro*, modulation of Spike abundance and processing in producer cells appears to reduce Spike incorporation into virions and diminish infectivity, while also attenuating recognition by anti-Spike antibodies, with possible implications for antibody-mediated effector functions (27).

Extracellularly, secreted ORF8 can behave as a virokine-like factor that amplifies inflammatory responses in myeloid and dendritic cells and, at the maternal-fetal interface, engages complement, providing plausible mechanisms for both heightened acute severity and tissue-specific immunopathology. Clinically, circulating ORF8 correlates with acute disease severity and mortality in hospitalized patients (31), and naturally occurring variations such as L84S, which attenuates ORF8-driven inflammatory signaling *in vitro*, further illustrate how single substitutions may modulate ORF8’s inflammatory activity and, potentially, its contribution to disease severity. Importantly, ORF8 antigen has been reported to persist in circulation in some individuals, even after viral RNA becomes undetectable (48). Patient-level evidence links circulating ORF8 to an inflammatory signature and shows that ORF8 can induce inflammatory responses in patient-derived cells (49). Whether this translates into a causal role in post-acute sequelae, however, remains unresolved. More broadly, studies of post-acute SARS-CoV-2 antigenemia have yielded heterogeneous results: circulating viral antigens have been associated with PASC symptoms in some cohorts (61), whereas a blinded two-year longitudinal study found that antigenemia could persist in a minority of individuals but was not associated with the number or type of persistent symptoms (62). Although these studies did not assess ORF8 specifically, they caution against inferring a pathogenic role from antigen persistence alone. Thus, whether ORF8 antigenemia correlates with PASC symptoms independently of structural antigen persistence, contributes directly to PASC/Long COVID pathology, or instead marks ongoing immune dysregulation and/or viral burden remains to be established.

A recurring theme across these functions is the apparent tension between ORF8-mediated suppression of type I IFN and its amplification of inflammation. These activities are not necessarily contradictory: they may map onto spatially distinct pools of ORF8. Intracellular ORF8, retained in the ER and secretory pathway of infected cells, is positioned to antagonize IRF3-dependent IFN induction at the site of replication, whereas secreted ORF8 may act on bystander myeloid and dendritic cells to amplify inflammatory cytokine programs. Under this proposed model, ORF8 may exert divergent effects on innate immunity depending on its localization and cellular context, with early IFN suppression favoring viral replication and later, secretion-driven inflammation potentially contributing to the immunopathology of severe disease. This framework is clinically relevant to post-infectious inflammatory syndromes such as multisystem inflammatory syndrome in children (MIS-C), a severe hyperinflammatory condition following SARS-CoV-2 infection. MIS-C has been associated with altered type I IFN activity, plasmacytoid dendritic cell depletion and a prominent inflammatory cytokine and chemokine signature (63), with additional reports highlighting concomitant dysregulation of inflammasome/IL-1β and type I IFN pathways in selected clinical contexts (64). Although a direct role for ORF8 in MIS-C has not been demonstrated, the overlap between these clinical features and the innate immune axes modulated by ORF8 underscores the potential pathological relevance of IFN-inflammasome imbalance and supports further investigation of SARS-CoV-2 accessory proteins in post-infectious inflammatory syndromes.

A general caveat applies to much of the mechanistic evidence reviewed here: most findings derive from overexpression systems, recombinant protein assays, or *in silico* docking studies, which may not faithfully recapitulate ORF8 biology at the protein levels and molecular contexts present in naturally infected cells. Systematic validation in primary human cells and authentic SARS-CoV-2 infection models, including physiologically relevant ORF8 concentrations and appropriate comparators, remains a priority before mechanistic conclusions can be translated to the clinical setting. Future research should include rigorous definition of (i) residue-level determinants for HLA-I loss, (ii) *bona fide* entry receptors and uptake routes in primary human cells, (iii) secretion kinetics and glycoforms across variants, and (iv) *in vivo* dose-response relationships that link circulating ORF8 levels to specific clinical endpoints. Addressing these gaps will clarify when and where ORF8 acts predominantly as an immune-evasion factor, an inflammatory amplifier, or both, and will better delineate its potential biomarker and therapeutic relevance in acute and post-acute COVID-19.
